# A Versatile Strategy for Production of Membrane Proteins with Diverse Topologies: Application to Investigation of Bacterial Homologues of Human Divalent Metal Ion and Nucleoside Transporters

**DOI:** 10.1371/journal.pone.0143010

**Published:** 2015-11-25

**Authors:** Cheng Ma, Zhenyu Hao, Gerard Huysmans, Amelia Lesiuk, Per Bullough, Yingying Wang, Mark Bartlam, Simon E. Phillips, James D. Young, Adrian Goldman, Stephen A. Baldwin, Vincent L. G. Postis

**Affiliations:** 1 Key Laboratory of Pollution Processes and Environmental Criteria (Ministry of Education), College of Environmental Science and Engineering, Nankai University, Tianjin, China; 2 Astbury Centre for Structural Molecular Biology, School of Biomedical Sciences, University of Leeds, Leeds, United Kingdom; 3 Krebs Institute for Biomolecular Research, Department of Molecular Biology and Biotechnology, University of Sheffield, Firth Court, Western Bank, Sheffield, United Kingdom; 4 College of Life Sciences, Nankai University, Tianjin, China; 5 State Key Laboratory of Medicinal Chemical Biology, Nankai University, Tianjin, China; 6 Research Complex at Harwell, Harwell Science and Innovation Campus, Didcot, Oxfordshire, United Kingdom; 7 Department of Physiology, University of Alberta, Edmonton, Canada; 8 Division of Biochemistry, Department of Biosciences, University of Helsinki, Helsinki, Finland; 9 Biomedicine Research Group, Faculty of Health and Social Sciences, Leeds Beckett University, Leeds, LS1 3HE, United Kingdom; University of Roskilde, DENMARK

## Abstract

Membrane proteins play key roles in many biological processes, from acquisition of nutrients to neurotransmission, and are targets for more than 50% of current therapeutic drugs. However, their investigation is hampered by difficulties in their production and purification on a scale suitable for structural studies. In particular, the nature and location of affinity tags introduced for the purification of recombinant membrane proteins can greatly influence their expression levels by affecting their membrane insertion. The extent of such effects typically depends on the transmembrane topologies of the proteins, which for proteins of unknown structure are usually uncertain. For example, attachment of oligohistidine tags to the periplasmic termini of membrane proteins often interferes with folding and drastically impairs expression in *Escherichia coli*. To circumvent this problem we have employed a novel strategy to enable the rapid production of constructs bearing a range of different affinity tags compatible with either cytoplasmic or periplasmic attachment. Tags include conventional oligohistidine tags compatible with cytoplasmic attachment and, for attachment to proteins with a periplasmic terminus, either tandem *Strep*-tag II sequences or oligohistidine tags fused to maltose binding protein and a signal sequence. Inclusion of cleavage sites for TEV or HRV-3C protease enables tag removal prior to crystallisation trials or a second step of purification. Together with the use of bioinformatic approaches to identify members of membrane protein families with topologies favourable to cytoplasmic tagging, this has enabled us to express and purify multiple bacterial membrane transporters. To illustrate this strategy, we describe here its use to purify bacterial homologues of human membrane proteins from the Nramp and ZIP families of divalent metal cation transporters and from the concentrative nucleoside transporter family. The proteins are expressed in *E*. *coli* in a correctly folded, functional state and can be purified in amounts suitable for structural investigations.

## Introduction

Membrane proteins account for 20–30% of the genes in most sequenced genomes [1,2]. They perform many functions essential to the life of cells, ranging from uptake of nutrients to information transfer, and their malfunction is implicated in a wide range of diseases [3]. In addition to their roles in normal physiology, they also represent approximately 60% of current therapeutic drug targets [4,5]. Gaining a detailed understanding of their molecular mechanisms requires high resolution structural information, but at present relatively few membrane protein structures have been solved: in May 2015 the Protein Databank included only about 1,654 membrane protein structures, compared to more than 90,000 structures of water-soluble proteins. In large part this reflects the difficulty of expressing and purifying these amphipathic proteins and maintaining them in a sufficiently stable state for biophysical and structural studies [6,7].

The majority of membrane protein structures to date are of prokaryotic origin, although an increasing number of structures have been determined for eukaryotic proteins expressed in yeast, insect or mammalian cells [6]. Although *E*. *coli* has proven to be a robust expression host for recombinant expression of prokaryotic membrane proteins [8], heterologous expression can result in poor expression levels or production of misfolded protein, either within the membrane or in the form of inclusion bodies [9,10]. A known source of such problems is the effect of added affinity tags on the membrane insertion and folding of the proteins, probably reflecting the importance of the charges present in the cytoplasmic and non-cytoplasmic regions flanking transmembrane segments in the correct biosynthesis of α-helical membrane proteins [11,12]. For example, we have shown that attachment of oligohistidine tags to the periplasmic termini of membrane proteins drastically impairs their production in *E*. *coli*, while similar attachment to cytoplasmic termini is well tolerated [13].

While N_in_-C_in_ topologies dominate the membrane proteomes of most organisms, one or both termini of a substantial fraction of membrane proteins are located on the extracellular/periplasmic side of the membrane [1]. A variety of approaches has been adopted for the production and purification of such proteins, including attachment of *Strep*-tag II sequences to periplasmic C-termini [13] and attachment of an N-terminal maltose binding protein (MBP) preceded by a signal sequence, enabling translocation of an oligohistidine tag across the membrane [14]. In a novel approach, Quick and Wright attached an additional transmembrane helix from glycophorin A to the C-terminus of the human sodium-linked glucose transporter hSGLT1 to bring its C-terminus to the cytoplasmic side of the membrane, allowing overexpression of functional, His-tagged protein in *E*. *coli* [15]. The same approach was more recently employed as a generic strategy to convert membrane proteins with a C_out_ topology to a C_in_ topology, allowing use of C-terminally attached GFP as a reporter of expression and folding [16]. However, for membrane proteins of unknown structure the location of the termini cannot usually be predicted with 100% accuracy, in part because the algorithms employed for prediction of topology typically do not take into account the substantial changes in protein folding that may occur subsequent to insertion of individual transmembrane segments into the membrane, and the effects of neighbouring helices on the insertion of a marginally hydrophobic transmembrane helix [17]. It is therefore usually necessary to test a variety of constructs, bearing different tags and employing different promoters, together with a range of expression strains, in order to identify conditions suitable for production of the amounts of correctly folded, functional protein required for structural and other studies.

To facilitate the rapid investigation of multiple expression conditions, we have previously described the use of autoinduction in deep well plates to optimise expression of constructs encoding membrane proteins [18]. In that study, the proteins tested had at least one cytoplasmic terminus, to which affinity tags were attached. To extend this approach to membrane protein families whose members possess extracytoplasmic termini, we describe here two new strategies for the rapid identification of constructs/conditions yielding good expression, whatever the topology of the original target of interest.

Our first strategy was to use bioinformatic analyses to identify transporters that differ from the majority of members of the cognate protein family in possessing an additional putative transmembrane segment, thus bringing either the N- or C-terminus of the protein to the cytoplasmic side of the membrane. Such proteins are compatible with attachment of standard affinity tags such as oligohistidine.

Our second strategy was to create a new family of expression vectors optimised for “out” expression. We have exploited restriction sites less commonly found in prokaryote genomic DNA to create a family of expression vectors between which open reading frames (ORFs) can readily be exchanged. In addition to tags previously employed [13,18], these vectors include ones, such as tandem *Strep*-tag II sequences and MBP preceded by a signal sequence, that are compatible with attachment to the extracytoplasmic termini of membrane proteins. The tighter binding of Streptactin to the tandem *Strep*-tag II than the single *Strep*-tag II [19] allows purification of membrane proteins to near homogeneity in a single step.

Our test cases were prokaryotic members of three families of membrane transporters of biomedical importance where one or both termini are typically extracytoplasmic. The families studied were: the concentrative nucleoside transporter (CNT) family of cation/nucleoside co-transporters [20] with an N_out_-C_out_ topology [21,22], and two families of divalent metal ion transporters: the “Zrt, Irt-like Protein” (ZIP) family, also with an N_out_-C_out_ topology [23], and the “Natural Resistance-associated Macrophage Protein” (Nramp) [24], with an N_in_-C_out_ topology. A total of five prokaryotic transporters representative of these families were successfully expressed in *E*. *coli* using these approaches and purified to near homogeneity in sufficient amounts and with a monodispersity in detergent solution necessary for subsequent biochemical investigations.

Assays showed that all but one of the expressed proteins were functionally active. Interestingly the two members of the ZIP family investigated proved to have channel-like properties with zinc ion, while the member of the Nramp family investigated, from *Enterococcus faecalis*, predicted to be an iron or manganese transporter [25,26], appears to be a zinc transporter. The strategies described in this study should prove generally useful for the production of membrane proteins with difficult topologies for biophysical and biochemical studies.

## Materials and Methods

### Materials

Genomic DNA for *Pseudomonas fluorescens* Pf-5 was obtained from ATCC, while genomic DNAs from *Enterococcus faecalis* strain V583 and *Vibrio cholerae* strain N16961 were kindly provided by the Department of Microbiology, University of Leeds, and by the University of York, respectively. Genomic DNA for *Achromobacter xylosoxidans* was prepared from strain NCIMB 11015, while the ORF of the ZIP family member CB-ZIP from *Coxiella burnetii* was synthesised by GenScript USA Inc. (Piscataway, NJ 08854, USA), following optimisation by the JCat method [27] for expression in *E*. *coli*. Phospholipids were obtained from Avanti Polar Lipids Inc. (Alabaster, AL, USA), while the detergents n-dodecyl-β-D-maltoside (DDM) and n-decyl-β-D-maltoside (DM) were from GLYCON Biochemicals GmbH (Luckenwalde, Germany) and lauryldimethylamine-N-oxide (LDAO) and octaethylene glycol monododecyl ether (C_12_E_8_) were from Anatrace (Maumee, USA). HisPur™ Cobalt resin was from Perbio Science UK Ltd. (Cramlington, UK) and Ni^2+^-NTA Agarose was from Generon Ltd. (Maidenhead, UK), while PD-10 desalting and Hitrap Q HP columns were from GE Healthcare (Little Chalfont, UK). Centrifugal concentrators were obtained from Sartorius Stedim Biotech. Plasmids pET26 and pCR®-Blunt were obtained from Novagen (Millipore (UK) Ltd.) and Invitrogen (Life Technologies Ltd., Paisley, UK) respectively. An N-terminally hexahistidine-tagged form of TEV protease, encoded by pTH24:TEV_SH_ [28] and an N-terminally octahistidine-tagged form of HRV-3C protease, encoded by a pET28 derivative, were purified by immobilised metal affinity chromatography (IMAC) following expression in *E*. *coli*. Strep-Tactin® Superflow® resin and Strep-tag® II specific monoclonal antibody were obtained from IBA GmbH (Goettingen, Germany) and HRP-labelled monoclonal anti-His-tag antibodies (Clone # AD1.1.10) from R&D Systems Europe Ltd. (Abingdon, UK). COmplete® EDTA-free protease inhibitor cocktail tablets were obtained from Roche Applied Science. [5,6-^3^H]Uridine (39.5 Ci/mmol) was obtained from PerkinElmer Life Sciences Ltd, UK and FluoZin™-1 from Life Technologies Ltd. (Paisley, UK).

### Expression trials

Expression trials were typically performed in four *E*. *coli* strains in parallel: BL21-gold(DE3) (Stratagene), BL21 Star^TM^ (DE3) (Invitrogen; [29]), C41(DE3) and C43(DE3) (Lucigen Corporation; [30]), all of which had been transformed with the plasmid pRARE2 (Novagen) to enhance translation efficiency of ORFs with codon usage different from endogenous *E*. *coli* genes [31]. Typically, autoinduction of 1 mL cultures in modified M9 medium (M9_auto_); Lysogeny broth (LB_auto_) or Superbroth medium (SB_auto_) containing 0.5% glycerol, 0.05% glucose and 0.2% lactose was performed at 37°C for 24 h in 24-deep-well plates as previously described [18]. Cell lysates were prepared in buffer containing 1% Triton X-100 and 0.1 mg mL^-1^ lysozyme and their protein concentrations measured using the bicinchoninic acid (BCA) assay (Perbio Science UK Ltd). Samples were then analysed by SDS-12% (w/v) polyacrylamide gel electrophoresis (SDS-PAGE) followed by staining with Coomassie Brilliant Blue R250 (Fluka) or by western blotting using either horseradish peroxidase-labelled monoclonal antibody against oligohistidine tags or the *Strep* tag II sequence as appropriate [18]. For quantification of His-tagged constructs, known amounts of a hexahistidine-tagged form of TEV protease [28] were included on the gel. Following incubation with SuperSignal® West Pico chemiluminescent substrate (Perbio Science UK Ltd), signals were detected and quantified using a GeneGnome Detection system and GeneTools software, respectively (Syngene Bio Imaging).

### Large scale expression and membrane preparation

For preparative scale membrane production cultures were grown in flasks on a 4 L scale, or in a fermenter on a 30 L scale, essentially as previously described [18], using the optimum combination of host strain and medium for each target, established as described above. Typically, cultures were autoinduced for 24 h at 37°C. In the case of CNT constructs, where autoinduction was less effective, cultures were instead induced with 0.5 mM isopropyl β-D-thiogalactoside (IPTG) when a OD_600nm_ value of 0.7 was reached, and then incubated for a further 3 h (for *Strep*-tagged constructs) or 6 h (for MBP-tagged constructs) at 37°C. Following harvesting by centrifugation, cells were suspended at a concentration of 5–6 mL/g wet weight in Tris-EDTA buffer (20 mM Tris, 0.5 mM EDTA, pH 7.4 at 4°C) containing protease inhibitor (cOmplete® EDTA protease inhibitor cocktail tablets, Roche Applied Science, 1 tablet per 50 mL solution). They were then disrupted at 30 Kpsi using a TS series 2.2 KW continuous cell disruptor (Constant Systems Ltd., UK) and membranes were prepared by centrifugation as previously described. The latter were stored at -80°C at a concentration of 20–40 mg/mL in 20 mM Tris-HCl, pH 7.4 at 4°C, or in phosphate-buffered saline (PBS; 10 mM Na_2_HPO_4_, 1.8 mM KH_2_PO_4_, 137 mM NaCl, 4 mM KCl, pH 7.4).

### Detergent solubilisation and purification of affinity-tagged transport proteins

All stages of detergent solubilisation and protein purification were performed at 4°C unless otherwise stated.

### Solubilisation trials

Solubilisation trials were performed using *E*. *coli* membranes, harbouring the desired target protein, at a concentration of 5 mg/mL in 100 μL solubilisation buffer (50 mM HEPES, pH 7.4, 150 mM NaCl, 5% (w/v) glycerol) containing 5 mM imidazole and 0, 0.33, 1, or 1.5% detergent (DDM, DM, LDAO or C_12_E_8_). Samples were gently mixed for 2 h and then centrifuged at 100,000 *g*_av_ for 1 h. Samples of the mixture taken before centrifugation and of the supernatant were then subjected to analysis by western blotting. Following identification of the optimum solubilisation conditions these were then used to purify the individual target proteins as follows. All steps were performed at 4°C.

### Purification of double Strep-tagged CNTs

For purification of *Strep*-tagged CNTs, membranes (140 mg protein) were suspended at a concentration of 2 mg/mL in buffer 8 (Table 1) containing cOmplete protease inhibitor cocktail and 1% DDM. After incubation for 1 h the mixture was centrifuged at 100,000 *g*_av_ for 1 h to remove insoluble material and then the supernatant was diluted with an equal volume of buffer 9 (Table 1). It was then incubated overnight with 0.5 mL Strep-Tactin® Superflow® resin, with gentle mixing. After transfer to a column, the resin was next washed with 5 mL Buffer 10 (Table 1) containing 0.05% DDM. Fractions (0.5 mL) were then collected during passage through the column of Buffer 10 (Table 1) containing 2.5 mM d-desthiobiotin and 0.05% DDM. Fractions containing eluted protein, as assessed by their A_280nm_ values, were pooled and dialysed against Buffer 10 containing 0.05% DDM to remove desthiobiotin.

**Table 1:** Buffers.

Buffer name	HEPES (mM)	Bis-Tris (mM)	MES (mM)	Tris (mM)	KPi (mM)	pH	NaCl (mM)	K_2_SO_4_ (mM)	EDTA (mM)	Glycerol (w/v; %)
Buffer 1	50					8.0	150			20
Buffer 2	50					7.4	150			5
Buffer 3		50				6.0				5
Buffer 4		50				6.0	1000			5
Buffer 5			50			6.0				5
Buffer 6		2	2			6.8				
Buffer 7		20	20			6.8		50		
Buffer 8				50		7.0	100		1	
Buffer 9				50		7.0	100		1	10
Buffer 10				50		7.0	100		1	5
Buffer 11					50	7.4	150			10
Buffer 12					50	6.0	300		10	20
Buffer 13				50		8.0	150		1	5
Buffer 14				50		8.0	150			
Buffer 15				20		7.9	300			20
Buffer 16	50					8.0	300			20
Buffer 17	50					8.0	300			5

### Purification and proteolytic cleavage of MBP-tagged CNTs

For purification of MBP-tagged CNTs, membranes (100 mg protein) were suspended at a concentration of 2 mg/mL in Buffer 11 (Table 1) containing cOmplete, EDTA-free protease inhibitor cocktail and 1% DDM. After incubation for 1 h the mixture was centrifuged at 100,000 *g*_av_ for 1 h to remove insoluble material and the supernatant was then incubated with 4.0 mL HisPur™ Cobalt Resin overnight with gentle shaking. After transfer to a column, the resin was next washed with 40 mL Buffer 11 containing 5 mM imidazole and 0.05% DDM. Fractions (4 mL) were then collected during passage through the column of Buffer 12 containing 0.05% DDM and those containing eluted protein, as assessed by their A_280nm_ values, were pooled.

To remove the MBP tag, His-tagged HRV-3C protease added to the pooled fractions at a molar ratio of protease to protein of 1:15, followed by dialysis overnight against Buffer 13 containing 1 mM DTT and 1 mM uridine. Following subsequent incubation for 3 h at 10°C the digestion mixture was dialysed against Buffer 14 and then passed through a column (4.0 mL) of Ni-NTA agarose to remove the protease and MBP. The purified protein was then concentrated to 2 mg protein/mL using a 100 kDa cut-off centrifugal filter.

### Purification of MBP-tagged CBZIP

For purification of MBP-tagged CBZIP, membranes (200 mg) were solubilised by incubation for 1 h at a final concentration of 5 mg/mL in Buffer 16 (Table 1) containing 10 mM imidazole, 1% DM and 1 tablet cOmplete® EDTA-free protease inhibitor cocktail per 50 mL.Following centrifugation at 110,000 *g*_av_ for 1 h to remove insoluble material the supernatant was incubated with 4 mL HisPur™ Cobalt Resin for 2 h.

After transfer to a column, the resin was next washed with 30 mL Buffer 16 containing 20 mM imidazole and 0.2% DM followed by 30 mL. of the same buffer containing 40 mM and 0.2% DM. Finally, the protein was eluted in batch with 12 mL solubilisation buffer 17 (Table 1) containing 200 mM imidazole and 0.2% DM. The eluted material was dialysed overnight against Buffer 17 containing 0.2% DM, concentrated to 1 mg protein/mL using a 100 kDa cut-off centrifugal filter.

### Purification of N-terminally His-tagged AXZIP

For purification of AXZIP, membranes (100 mg) were solubilised by incubation for 1 h at a final concentration of 5 mg/mL in Buffer 16 (Table 1) containing 10 mM imidazole, 1.0% DM and 1 tablet cOmplete® EDTA-free protease inhibitor cocktail per 50 mL. Following centrifugation at 110,000 *g*_av_ for 1 h to remove insoluble material the supernatant was incubated with 2.0 mL HisPur™ Cobalt Resin for 2 h with gentle shaking. The unbound material was then removed by passage through a column with a filter and then the resin washed by passage of 30 mL Buffer 16 containing 10 mM imidazole and 0.2% DM and then with 30 mL portions of Buffer 15 containing 20 mM and 40 mM imidazole respectively. Finally, the resin was eluted with 12 mL Buffer 15 containing 0.2% DM and 200 mM imidazole. The eluted material was dialysed overnight against Buffer 17 (Table 1) containing 0.2% DM and then concentrated to 1 mg protein/mL using a 100 kDa cut-off centrifugal filter.

### Purification of C-terminally His-tagged MntH2

For purification of MntH2, membranes (300 mg) were solubilised by incubation for 1 h at a final concentration of 5 mg/mL in Buffer 1 (Table 1) containing 10 mM imidazole (pH 8.0), 1.5% DDM and 1 tablet cOmplete® EDTA-free protease inhibitor cocktail per 50 mL. Following centrifugation at 110,000 *g*_av_ for 1 h to remove insoluble material the supernatant was incubated with 16 mL HisPur™ Cobalt Resin for 2 h with gentle shaking. The unbound material was then removed by passage through a column with a filter and then the resin washed by passage of 160 mL Buffer 1 containing 20 mM imidazole and 0.05% DDM and then with 96 mL of Buffer 1 containing 40 mM and 80 mM imidazole respectively. Finally, the resin was eluted with 48 mL Buffer 2 (Table 1) containing 0.05% DDM and 200 mM imidazole. The eluted material was dialysed overnight against Buffer 3 (Table 1) containing 0.05% DDM, concentrated to 3 mg protein/mL using a 100 kDa cut-off centrifugal filter, then further purified by anion exchange chromatography on a 1 mL Hitrap Q HP column (GE Healthcare). After application of the sample the column was eluted with a gradient (20 mL) of 0–100% Buffer 4 (Table 1) containing 0.05% DDM. The peak fractions of eluted material, as judged by their A_280nm_ values, were combined, dialysed against Buffer 5 (Table 1) containing 0.05% DDM, concentrated again to about 10 mg/mL, flash frozen in liquid nitrogen and then stored at -80°C.

### Transport assays

Measurements of uridine uptake by IPTG-induced *E*. *coli* cells harbouring vectors encoding CNTs were performed at 25°C as described previously for the bacterial nucleoside transporter NupG [32]. [5,6-^3^H]Uridine was used at a final concentration of 50 μM and at a specific radioactivity of 1–5 mCi/mmol.

Assays of zinc uptake into reconstituted proteoliposomes harbouring ZIP or Nramp family transporters were performed essentially as described by Chao and Fu [33]. In brief, small unilamellar liposomes (50 mg/mL) were prepared from *E*. *coli* polar lipids by sonication in Buffer 6 (Table 1) containing 2 mM 2-mercaptoethanol (2-ME). A reconstitution mixture (1 mL) was then prepared by mixing 6.5 mg liposomes with 0.2–0.4 mg purified transporters plus sufficient n-octyl-β-D-glucoside (β-OG) to give a final concentration of 1% (w/v) in Buffer 7 (Table 1) containing 2 mM 2-ME. Following incubation at 20°C for 20 min to ensure complete solubilisation of lipids, reconstitution of proteoliposomes was achieved by passage through a PD-10 desalting column, pre-equilibrated with Buffer 7 (Table 1). The cloudy void volume fraction was centrifuged at 140,000 *g*_av_ for 45 min to pellet the proteoliposomes, which were then resuspended in 200 μL Buffer 7. Control liposomes were prepared following exactly the same procedure but without addition of protein. The proteoliposomes and control liposomes were then loaded with the Zn^2+^-sensitive indicator FluoZin™-1 at a final indicator concentration of 200 μM by bath sonication (10 s), freeze-thawing in liquid nitrogen and then an additional 10 s sonication. Proteoliposomes containing entrapped indicator were then separated from free indicator by passage through a second PD-10 desalting column, pre-equilibrated with Buffer 7.

Kinetic experiments were performed in fluorescence mode on a stopped-flow apparatus (Applied Photophysics Ltd., Leatherhead, UK) at 20°C. Proteoliposome samples and Buffer 7 containing the desired concentration of ZnCl_2_ (0–4 mM) were loaded into two separate mixing syringes of equal volume, and transport reactions were initiated by pushing 1 mL of fresh reactants at a 1:1 ratio through the 100 μL mixing cell. For measurements of fluorescence changes, samples were excited at 490 nm, and emission was monitored using a filter with a cut-off wavelength of 515 nm. The base-line fluorescence was set by flushing reactants through the mixing cell and adjusting the photomultiplier tube voltage to a level that would give a 1 V signal in the middle of the -5 –+5 V detectable range. The *K*_d_ for zinc binding to FluoZin™-1 is 8 μM, such that at the concentration of indicator employed (200 μM) there will be an approximately linear fluorescent response (ΔF) to the total intravesicular zinc concentration. The fluorescence response ΔF was normalised to the maximum fluorescence change ΔF_max_ obtained upon release of the encapsulated indicator to 2 mM Zn^2+^ by addition of 1% β-OG, representing the total vesicle loading with FluoZin™-1. This yielded the dimensionless fluorescence change ΔF/ΔF_max_, which was plotted as a function of time.

### Circular dichroism spectroscopy

Circular Dichroism (CD) spectroscopy measurements were typically performed at 20°C using a Chirascan CD spectrometer (Applied Photophysics Ltd.) with continuous nitrogen purging. Samples (0.25–0.5 mg/mL) in the corresponding stocking buffers as mentioned above were analysed in Hellma quartz cuvettes of path lengths 0.1 or 0.01 cm. Each spectrum represented the average of at least 2 scans from 180 nm to 260 nm. Spectra were recorded at a speed of 1 nm/s, bandwidth 1 nm. CD data were analyzed using DichroWeb [34,35]. To examine their thermal stability, CD spectra from 180 nm to 260 nm were recorded at a speed of 1 nm/0.3s during the heating of purified proteins from 10°C to 90°C at a rate of 1°C per min. To estimate the melting temperature, the resultant data were fitted to the Boltzmann equation for a sigmoid curve using Origin8 (OriginLab corporation, Northampton, USA).

## Results

### Rationale behind target selection

We have previously demonstrated that attachment of N- or C-terminal oligohistidine tags is the best approach for expressing and purifying prokaryotic membrane proteins in *E*. *coli* [13] as long as both the N- and C-terminus are cytoplasmic. In this study, we specifically chose three protein families where one or both termini are predicted to be periplasmic, and used BlastP searches of the UniProt protein sequence database to ascertain whether any family members possessed topologies more suitable for tagging, *i*.*e*. where the termini were located on the cytoplasmic side of the membrane.

**Fig 1:**
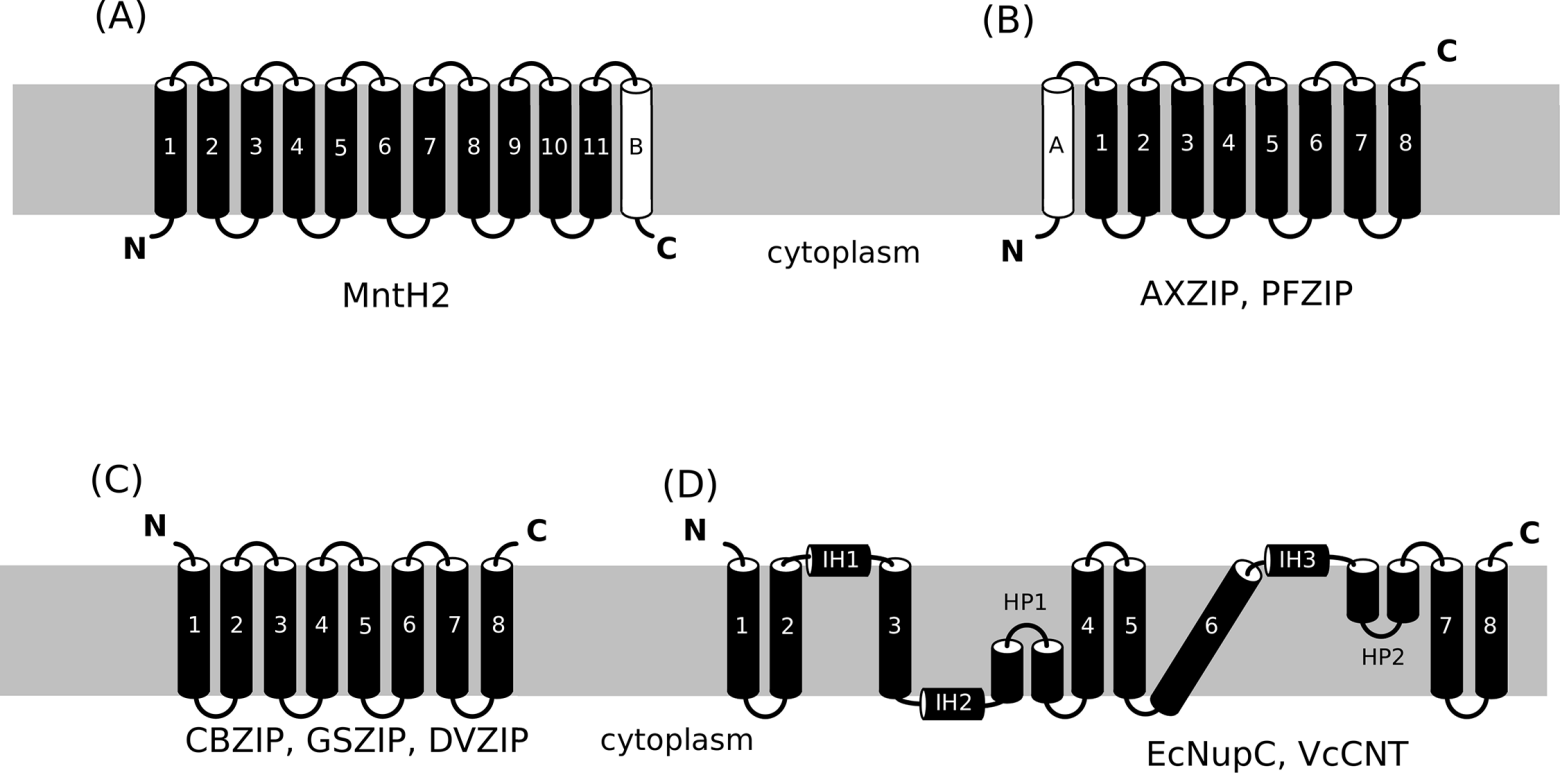
Putative membrane topology of studied transporters: (A) MntH2 from the Nramp family, (B) AXZIP, PFZIP from the ZIP family, (C) CBZIP, GSZIP, DVZIP from the ZIP family and (D) EcNupC, VcCNT, from the CNT family investigated in the present study. The TMs of the human homologues are shown in black. The putative additional TM helixes are shown in white.

While eukaryotic members of the Nramp family have an N_in_-C_in_ topology with 12 TM segments [24], the *E*. *coli* member MntH has only 11 TM segments such that the C-terminus is located on the extracellular/periplasmic side of the membrane [36], as do most of the other prokaryotic Nramp family members identified by BlastP searches. However, an Nramp from Lactobacillales had an additional hydrophobic, poorly-conserved region near the C-terminus that was predicted to be membrane-spanning using the TMHMM2.0 [1], PHOBIUS [37] and TOPCONS [38] programs (Fig 1A). This would place the C-terminus on the cytoplasmic side of the membrane. We chose one such protein for investigation, MntH2 from *Enterococcus faecalis* (UniProt accession Q836Q1).

For the Zip family, almost all prokaryotic and eukaryotic family members are predicted to possess an 8TM, N_out_-C_out_ topology [22] (Fig 1C), which has been experimentally confirmed for human ZIP13 [39]. Our BlastP searches identified a few members of the family from the Proteobacteria, in the GufA subfamily (S1 Fig), that possessed an additional hydrophobic, poorly-conserved region near the N-terminus. Sequence analysis [1,37,38] again suggested that this was membrane-spanning (Fig 1B) but that it was not a signal sequence, unlike in some eukaryotic family members [23]. The N-termini of the proteins would be located on the cytoplasmic side of the membrane. We thus chose two such proteins, one from *Pseudomonas fluorescens* (UniProt accession Q4K7I2), PFZIP, and one from *Achromobacter xylosoxidans* (UniProt accession V9S2P1), AXZIP. We also included a third prokaryotic ZIP protein, from *Coxiella burnettii* (CBZIP), for two reasons. First, it belongs to ZIP subfamily II, as opposed to ZIP subfamily I, and thus constitutes a good model for investigation of structure/function relationships in this group of transporters (Fig 1C). This is the most common of the ZIP subfamilies in mammals. Second, like all other ZIP subfamily II members, it is predicted to have the most difficult topology: N_out_-C_out_, thus providing the most stringent test of our new vector system.

Eukaryotic members of the CNT family have an N_in_-C_out_ topology, while their prokaryotic counterparts lack three N-terminal TM helices and so possess an N_out_-C_out_ topology [21,22,40] (Fig 1). Despite extensive searches, we did not find any prokaryotic CNTs with other topologies. We therefore chose two prokaryotic targets (Fig 1D): VcCNT, a sodium-dependent nucleoside transporter from *Vibrio cholera*, and NupC, a proton-dependent nucleoside transporter from *E*. *coli*, the function of which has been well characterised but for which no structural information is currently available [41,42]. These proteins have about 25% sequence identity to their human counterparts, hCNT1-3 [22].

MntH2, AXZIP and PFZIP thus represent our model proteins for expression and purification of membrane proteins with at least one terminus in the cytoplasm, while CBZIP, NupC and VcCNT have been chosen as examples of expression and purification of membrane proteins with both termini in the periplasm.

### Generation of expression vectors

**Fig 2:**
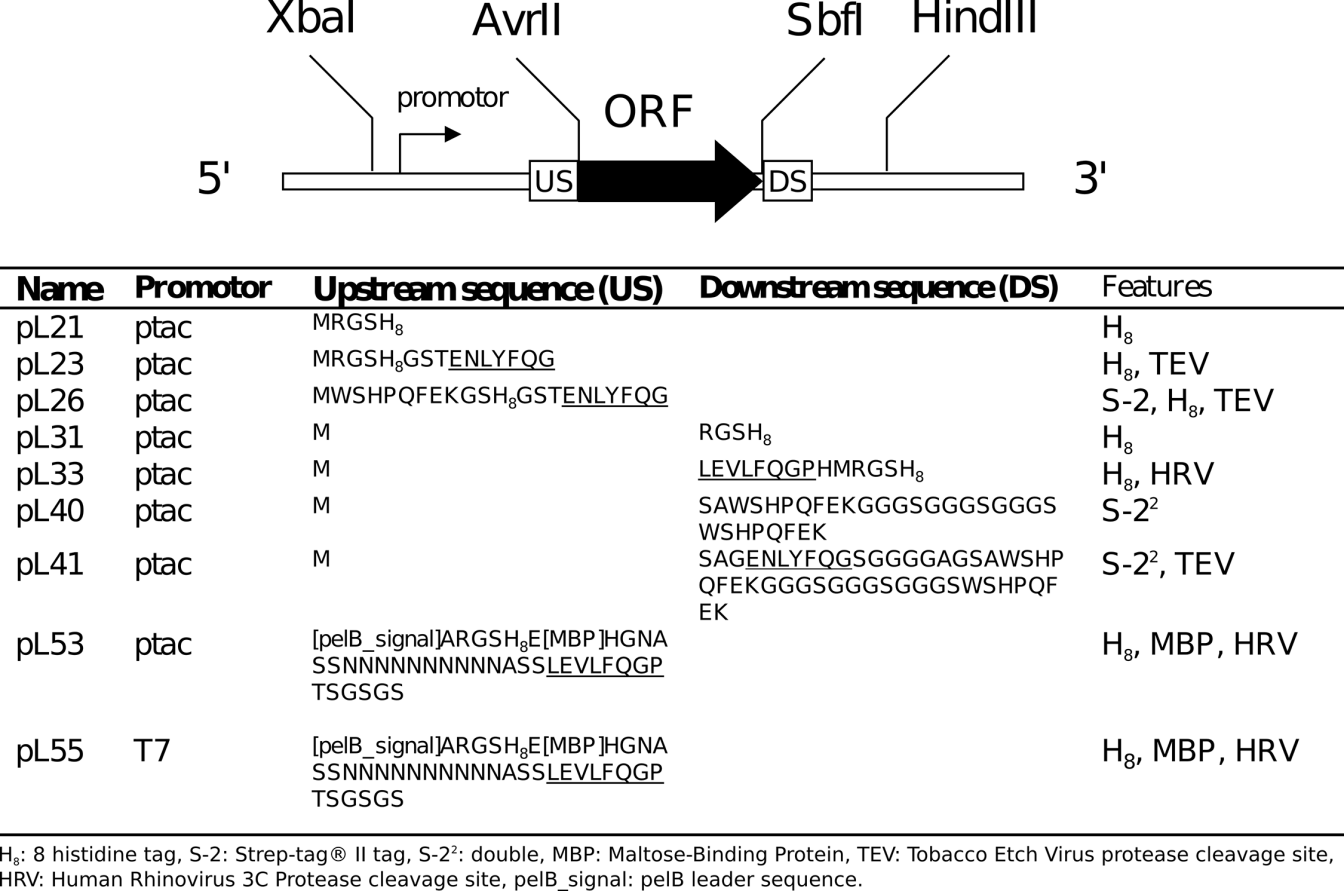
Schematic representation of the constructs used in this study is depicted in the panel above. The summary of the constructs details is shown in the table of the panel below.

To facilitate the rapid screening of expression constructs bearing different affinity tags, and to compare the use of different promoters, we cloned target ORFs, flanked by suitable restriction sites, into the vector pCR-Blunt® (Invitrogen) to generate entry vectors. Restriction sites chosen were *Avr*II and *Sbf*I, because these occur relatively infrequently in many bacterial genomes and so are less commonly found within target ORFs than, for example, *Eco*RI sites. If sites are endogenously present within the ORFs, a number of compatible sites are available as alternatives in each case. Primers used for amplification of the targets used in the present study, and for subsequent DNA sequencing (Source BioScience, Nottingham, UK) to verify the sequences of the ORFs and flanking regions in entry and expression vectors, are listed in S1 Table. The target ORFs were then subcloned into a range of expression vectors (Fig 2) bearing the same or compatible restriction sites, which facilitated not only transfer from the entry vector but exchange of ORFs between different expression constructs. Two types of expression vectors were generated for the present study, using as a backbone either pTTQ18, in which expression is under the control of the *tac* promoter [43], or a pET vector which employs a T7 promoter [44]. A subset of the vectors constructed for the present project include cleavage sites for the highly selective Tobacco Etch Virus (TEV) protease or Human Rhinovirus 3C (HRV-3C) protease, enabling tag removal prior to crystallisation, if necessary. In addition, inclusion of such sites enables a second purification step following cleavage, for example to remove endogenously His-rich *E*. *coli* proteins such as AcrB [45]. In contrast to TEV protease, which we previously exploited [18], HRV-3C protease retains substantial activity at 4°C [20] and so is ideal for cleavage of temperature-sensitive membrane proteins in detergent solution.

### Expression and purification of transporters with at least one cytoplasmic terminus

#### The Nramp family transporter MntH2: both termini in

**Fig 3:**
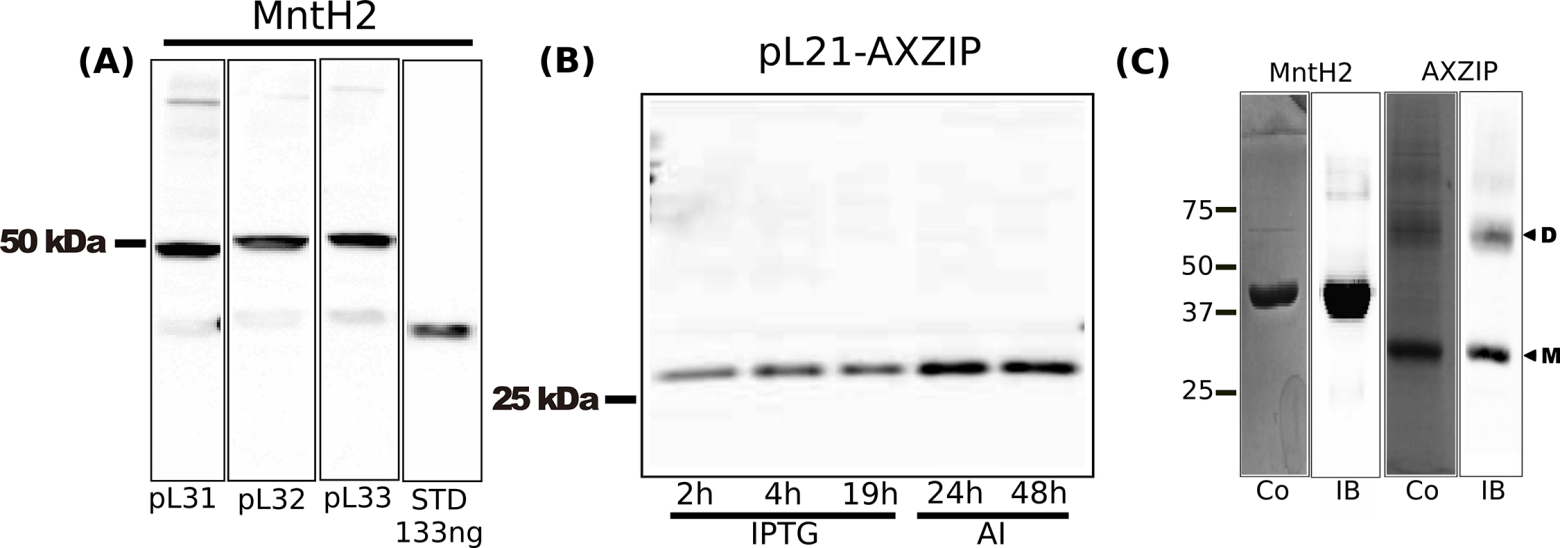
(A) Western blot analysis of expression levels yielded by 3 different His-tagged MntH2 constructs. The indicated constructs in *E*. *coli* strain BL21 Star™ (DE3) cells harbouring pRARE2 were autoinduced for 24 h as detailed in the text and then cell lysates analysed by SDS-PAGE/western blotting. Each lane contained 20 μg total protein. The amounts of His-tagged TEV protease standards blotted in parallel are indicated. The blot was stained for the presence of oligohistidine tags with HRP-labelled monoclonal anti-6 × polyhistidine antibody. The mobilities of marker proteins of known molecular mass are shown on the left. (B) Western blot analysis of samples from expression trials performed using pL21-AXZIP. Expression was performed in *E*. *coli* strains BL21Star either with IPTG induction (IPTG) or autinduction for amount of times indicated under the figure. The blot was stained with HRP-labelled monoclonal anti-6 × polyhistidine antibody. (C) Purification of MntH2 and AXZIP. Purified proteins were loaded on a gel and either stained with Coomassie Blue (Co) or transferred on a nitrocellulose membrane and stained with anti-His antibody (IB).

As described above, MntH from *E*. *faecalis* is predicted to have a 12 TM, N_in_-C_in_ topology (Fig 1). It is thus a better model for eukaryotic members of the family, which exhibit a similar 12 TM topology. The predicted “additional” 12^th^ TM segment should allow use of a conventional His tag. To test this, we made three constructs: with a C-terminal RGSH_8_ tag alone (pL31-MntH2), with a TEV cleavage site (pL32-MntH2), or with an HRV-3C (pL33-MntH2) cleavage site. Preliminary expression trials (data not shown) on pL31-MntH2 were consistent with C_in_ topology: autoinduction in *E*. *coli* strain BL21 Star™ (DE3) in SB medium for 24 h yielded good expression, and so these conditions were used to compare the expression of the three constructs. Western blots of induced cell lysates stained by antibodies against the His-tag revealed a major band of size ~ 50 kDa in each case (Fig 3A). The difference in the mobility of this band and the predicted sizes of the tagged proteins (60.3, 61.5 and 61.4 kDa respectively) likely reflects the anomalous mobility of integral membranes on SDS-PAGE [46].

#### ZIP family proteins with only N-terminus in

*P*. *fluorescens* PFZIP and *A*. *xylosoxidans* AXZIP are predicted to have a 9TM, N_in_-C_out_ topology, so it should be possible to attach a His-tag to the cytoplasmic N-terminus. We therefore made constructs encoding proteins with different N-terminal tags: RGSH_8_ (pL21-PFZIP and pL21-AXZIP), *Strep*-tag II sequence and a GSH_8_ tag plus a TEV cleavage site (pL26-PFZIP and pL26-AXZIP) (Fig 2). Expression trials on these showed very high levels of expression (~ 15 mg/L culture) using pL26-PFZIP following 24 h auto-induction at 37°C in SB medium in the C43(DE3):pRARE2 *E*. *coli strain* (data not shown). However, we were unable to solubilise it in any of the detergents tested (DDM, DM, LDAO or C_12_E_8_) and so work on PFZIP was discontinued. It is possible that the high levels of expression and the lack of solubilisation are linked: *i*.*e*. PFZIP is denatured.

With AXZIP, the highest levels of expression (~2.6 mg/L culture) came from pL21-AXZIP using 24 h auto-induction at 37°C in LB medium in *E*. *coli* BL21 Star (DE3):pRARE2 (Fig 3A).

Both the MntH2 variants and pL21-AXZIP could be successfully purified by IMAC on cobalt resin, MntH2 in DDM, yielding 1–2 mg protein/L; and pL21-AXZIP in DM, yielding ~ 0.5 mg protein/L. All yielded a single major Coomassie Blue-staining band on SDS-PAGE of the appropriate molecular mass (Fig 3C). With AXZIP, there were less intense bands of apparent sizes ~60 kDa and 90 kDa (Fig 3C) that, based on western blotting, appear to correspond to monomeric, dimeric and trimeric forms of the protein. Purification of AXZIP to homogeneity was thus successful.

**Fig 4:**
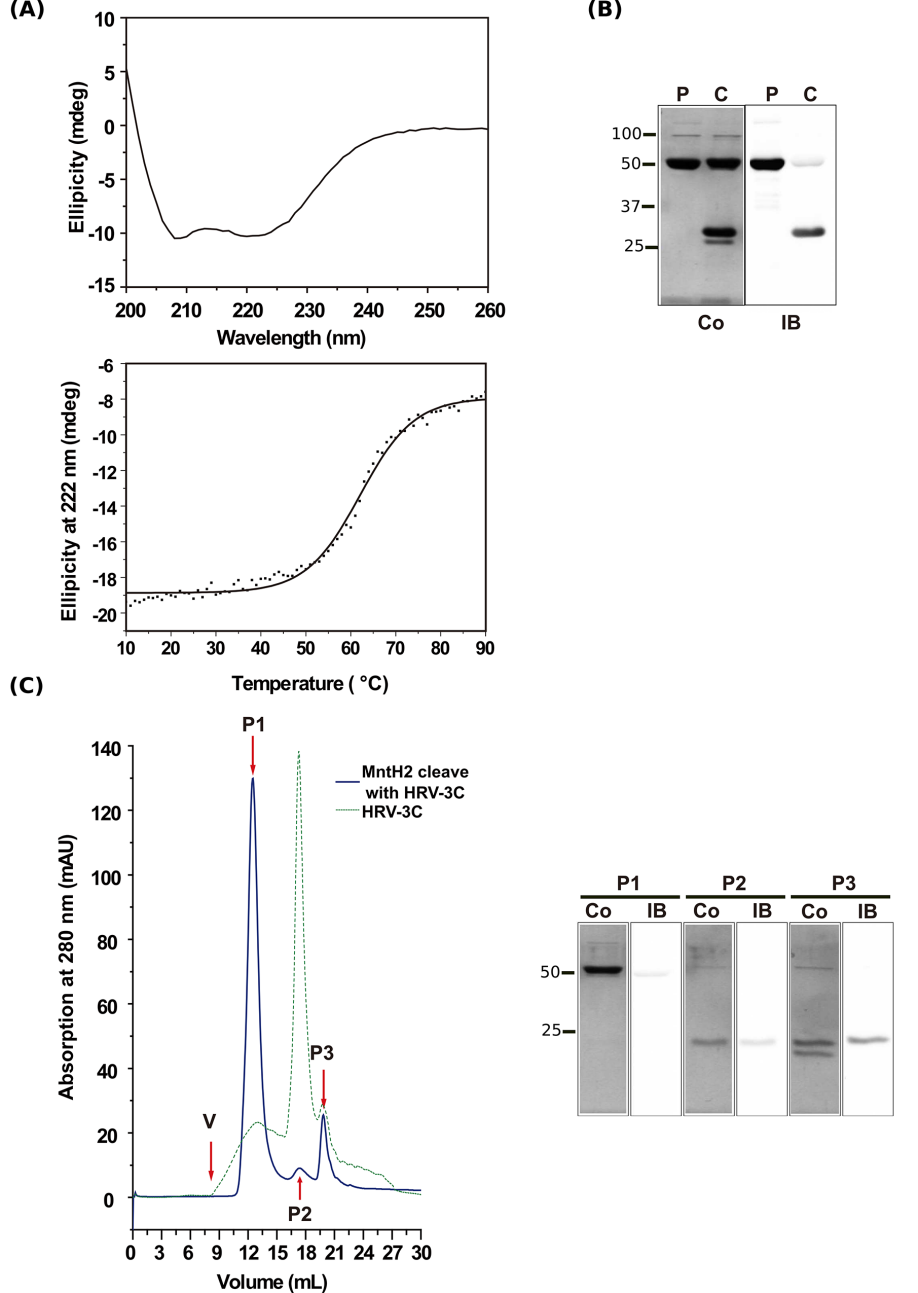
(A) Analysis of the secondary structure of His-tagged MntH2, expressed from construct pL33-MntH2, by circular dichroism spectroscopy (top pannel). The analysis of the spectrum using the SELCON3 algorithm indicated that the composition were 81.0% ± 0.2% α-helix, 0.5% ± 0.3% β-sheet and 21.4% ± 0.7% turns plus random coil. The thermal stability of MntH2 (Bottom pannel). IMAC purified pL33-MntH2 (0.125 mg/mL) was dissolved in 50 mM MES buffer, pH 6.0 containing 5% glycerol and 0.05% DDM. Thermal stability was measured by ramping the temperature from 10°C to 90°C, at a rate of 1°C per second. CD measurements were performed at 222 nm following each 1°C increase in temperature, and are plotted following correction for the buffer blank. The solid line shows a non-linear fit of these data to the Boltzmann equation. (B) Purification steps of MntH2. Pure MntH2 protein before (P) or after cleavage with HRV3C (C) was loaded on a gel and either stained with Coomassie Blue (Co) or transferred on a membrane and immunoblotted with anti-His antibody (IB). (C) Separation of HRV-3C protease-cleaved MntH2 from the protease by size-exclusion chromatography. Left panel: A_280nm_ profiles of the HRV-3C protease-cleaved MntH2 on a Superdex 200 10/300 column (“MntH2 cleaved with HRV”, solid blue line) and of HRV-3C protease alone (“HRV”, dotted green line). Right panel: Samples containing 20 μg of MntH2 before and after cleavage with HRV-3C protease, and of the indicated peak fractions from the elution profile (40 μL) were subjected to SDS-PAGE and then either stained with Coomassie blue (top) or transferred to nitrocellulose membrane and stained for the presence of oligohistidine tags with HRP-labelled monoclonal anti-6× polyhistidine antibody (bottom). The mobilities of marker proteins of known molecular mass (kDa) are shown on the left.

Conversely, some of the MntH2 preparations contained a minor contaminant of apparent size ~100 kDa (Fig 4B), which is probably due to the endogenous membrane protein AcrB [45]. To remove it, pL32-MntH2 protein was treated for 18 h at 4°C with TEV protease at molar ratios of protease: MntH2 of up to 2.5:1. However, western blotting revealed that the His tag remained attached to the protein (data not shown). In contrast, incubation of the pL33-MntH2 protein with 2 equivalents of HRV-3C protease yielded essentially complete cleavage after 24 h (Fig 4B). Surprisingly, the cleaved protein was retained on the IMAC column during re-chromatography (data not shown), but it could be separated from uncleaved MntH2 protein by SEC on a Superdex 200 column, in which the cleaved protein migrated as a single, symmetrical included peak indicative of monodispersity (Fig 4C). SEC-MALLS analysis of the uncleaved protein gave a molecular mass of 65.4 kDa, consistent with it being a monomer in detergent solution (data not shown).

#### Protein characterisation and activity measurements for MntH2 and AXZIP

We used two approaches to determine if the purified proteins were in their native folded state: their CD spectra and melting curves, and activity measurements. For both MntH2 (Fig 4A) and AXZIP (data not shown), CD spectra revealed the presence of two major negative bands, at ~ 208 nm and ~ 220 nm, indicative of α-helical structure [47]. The program SELCON3 (Sreerama and Woody 1993) predicted that MntH2 was 81.0% ± 0.2% α-helix, and 21.4% ± 0.7% turns plus random coil, as expected for a membrane protein. Heating MntH2 resulted in progressive loss of α-helical structure at increasing temperatures (Fig 4B), yielding an estimated melting temperature of 62.1 ± 6.0°C.

**Fig 5:**
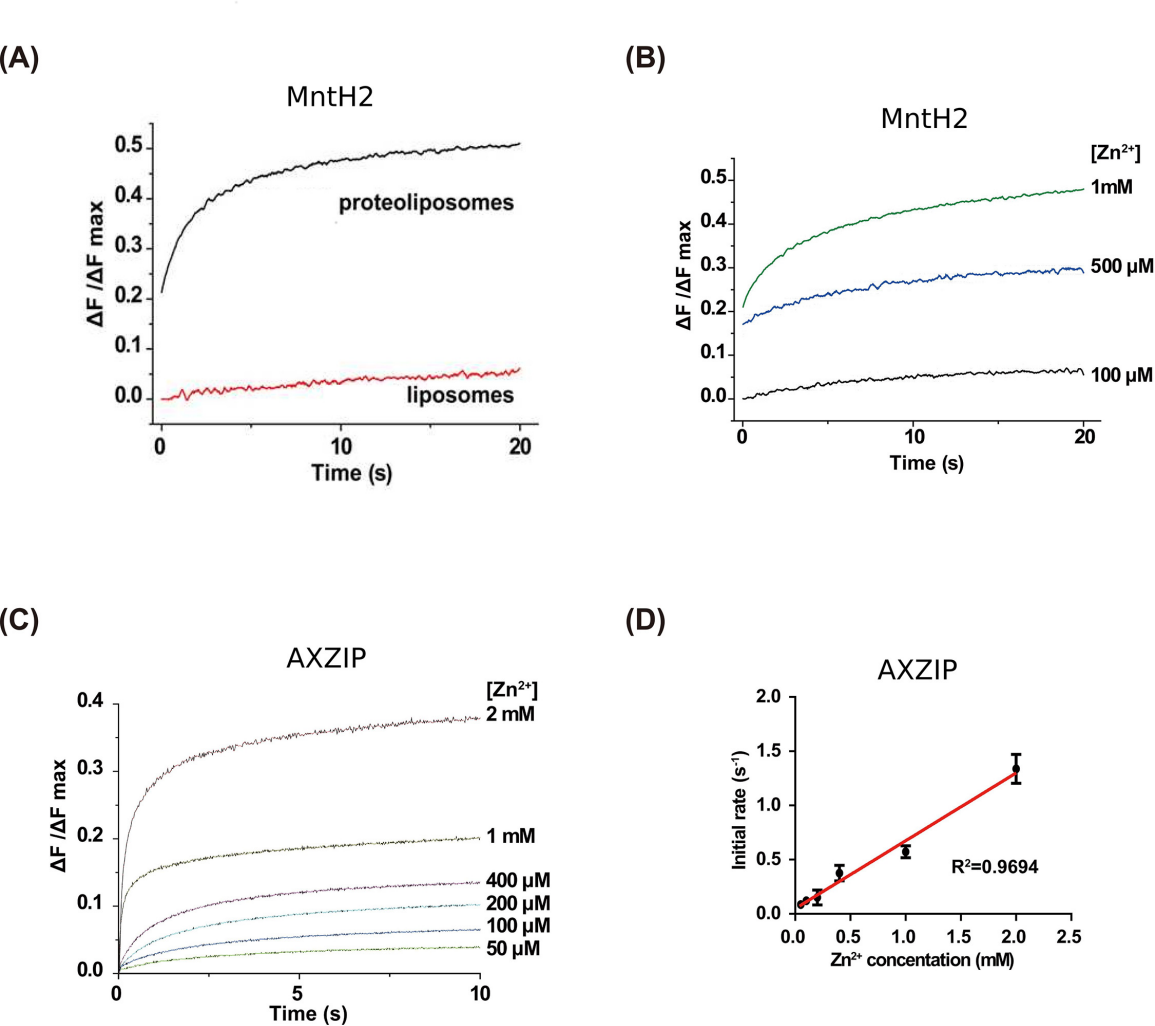
Comparison of zinc uptake by protein-free liposomes and proteoliposomes containing either MntH2 or AXZIP. (A) Liposomes and MntH2-containing proteoliposomes containing 200 μM FluoZin™-1 were mixed with zinc-containing assay buffer to yield a final [Zn^2+^] of 2 mM, and the resultant fluorescence changes recorded using a stopped flow fluorimeter. The normalized fluorescence change (ΔF/ΔF_max_) was determined by dividing the observed fluorescence change (ΔF) by that induced by adding 1% β-OG to the extravesicular medium (ΔF_max_). The results shown are the means of three measurements. (B) Fluorescent assays of zinc uptake were performed as described in the legend above, except that the extravesicular concentration of Zn^2+^ was varied from 100 μM to 1 mM. (C) same as in (B) but with AXZIP proteoliposomes and a Zn^2+^ concentration was from 50 μM to 2 mM (D) Initial rates of zinc uptake versus the extravesicular zinc concentration was subjected to linear regression. Initial rates (s^-1^) were estimated by fitting the first five data points (0–0.1 s) for each Zn^2+^ concentration in (C). All the results shown are the means of three measurements ± standard deviation.

We reconstituted purified MntH2 and AXZIP into proteoliposomes and used quenching of cation-specific fluorophores (see Materials and Methods) to measure their transport of divalent cations. MntH2 did not transport Mn^2+^ (data not shown), based on quenching of encapsulated calcein, which has been used to measure Mn^2+^ transport [48]. However, as other members of the Nramp family transport various divalent metal cations, including Zn^2+^ [49,50], we tested Zn^2+^ transport using the Zn^2+^-sensitive fluorescent dye “FluoZin™-1”. Proteoliposomes containing purified MntH2 took up Zn^2+^ much faster than protein-free liposomes, and the rate depended on Zn^2+^ concentration (Fig 5A). Zn^2+^ uptake was too rapid, even at 4°C, to allow accurate estimation by stopped flow fluorimetry of the initial rate of uptake, precluding determination of the kinetic parameters of transport (Fig 5B).

In AXZIP-containing proteoliposomes, the rise in ΔF/ΔF_max_ with time (data not shown) is much faster than for control proteoliposomes, indicating that AXZIP is specific for Zn^2+^ uptake. The initial rate of fluorescence change had a linear dependence on extravesicular zinc concentration, with no evidence of saturation up to the maximum concentration investigated (2 mM) (Fig 5C and 5D), suggesting that AXZIP, like ZIPB, may be a Zn^2+^ channel [51].

### Expression and purification of transporters where both termini are non-cytoplasmic

Both termini being non-cytoplasmic precludes the use of simple His-tags. We therefore adopted two tagging strategies: first, as a single periplasmic *Strep*-tag II is compatible with high level expression of *E*. *coli* MntH2 [13], we added tandem *Strep*-tag II sequences to the C-termini of the proteins (expression vectors pL40 and pL41). Second, we added a His-tag to the N-terminus of the proteins between the MBP and the signal sequence (expression vector pL53), so that the N-terminus is correctly directed to the periplasmic side of the membrane. We tested these new vectors for CBZIP and for two CNT family transporters, all of which have N_out_-C_out_ topologies.

**Fig 6:**
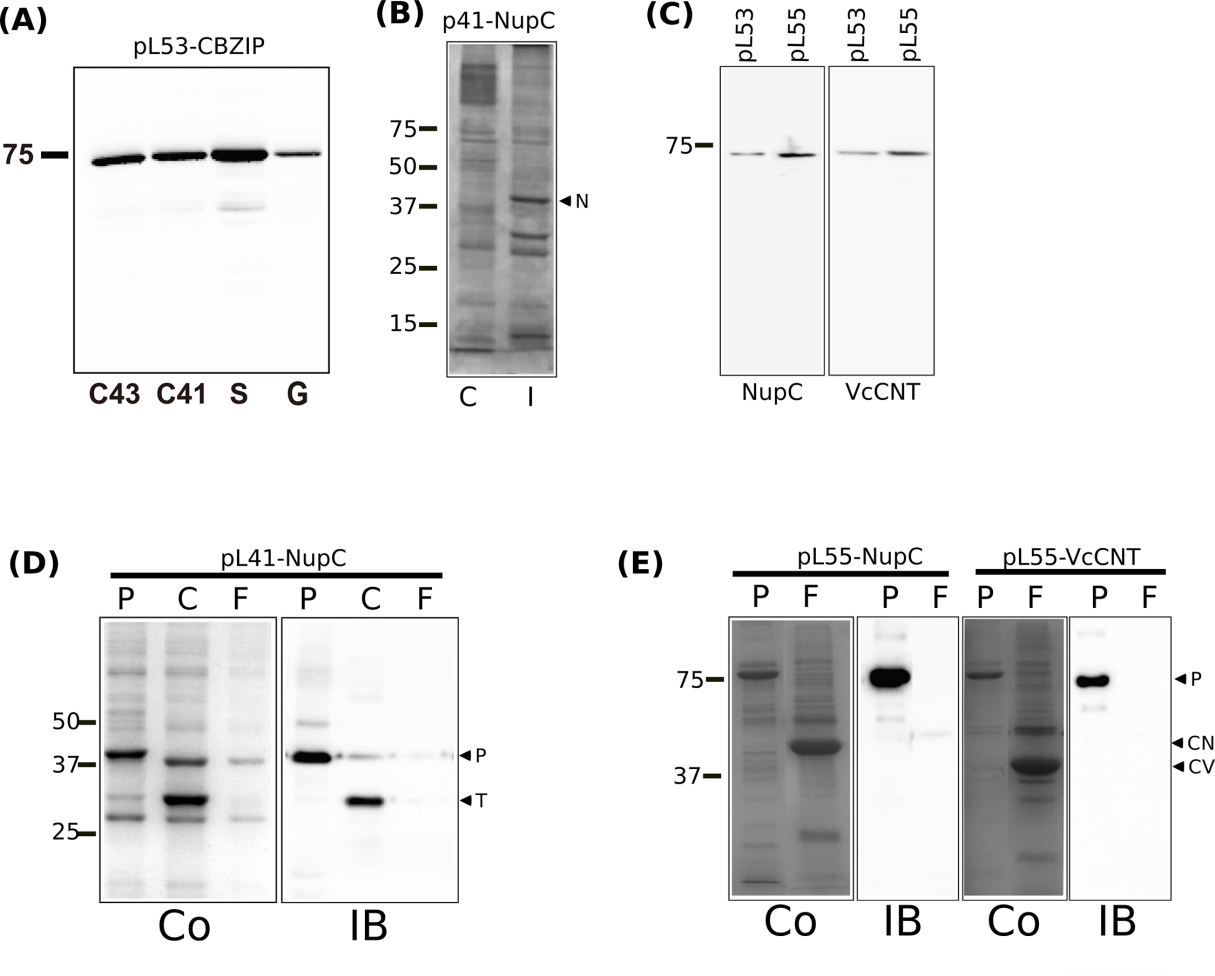
(A) Expression tests of pL53-CBZIP in various strains: C41, C43, BL21 Star (S) or BL21 Gold (G). Total extracted were loaded on a gel and stained with Coomassie Blue. (B) pL53-CBZIP was expressed in BL21 Star (S) and purified with an affinity column. Pure CBZIP was loaded on a gel and stained with Coomassie Blue (Co) or transferred onto a membrane and immunoblotted with Anti-His antibody (IB). (C) Expression test of p40-NupC. Cells expressing NupC were induced with IPTG. After 3h induction, cells were lysed by water-lysis technique as described in material and methods, loaded on a gel and stained with Coomassie Blue (I). Uninduced cells were loaded as a control (C). (D) Purified NupC protein was loaded on gel before (P) and after cleavage by TEV (C), and submitted to a negative purification to remove His-TEV and collect NupC in the flow through (F). Comparison between Coomassie Blue (Co) stained and immunoblot (IB) with antiHis Antibody is shown. The size of the purified protein (P) or the TEV protein (T) is shown on the right. (E) Effect of promoter on expression of NupC and VcCNT. Whole cellular extracts expressing NupC or VcCNT on either a pL53 (ptac promoter) or pL55 vector (T7 promoter). (F) Purification of NupC and VcCNT as described in (D).

Initial attempts at purification of CBZIP employed the construct pL40-CBZIP, which has two tandem *Strep*-tag II sequences at the C-terminus. However, while this construct could successfully be expressed at a level of ~ 3.4 mg/L culture following autoinduction for 24 h at 37°C in SB medium using *E*. *coli* strain C43(DE3): pRARE2 as host, we could not solubilise and purify it. pL53-CBZIP, with the N-terminal MBP-His_8_ tag (Fig 2) yielded up 14 mg/L culture by autoinduction for 24 h in LB medium in *E*. *coli* BL21 Star™ (DE3) pRARE2 (Fig 6A). The tagged protein could be successfully purified by IMAC on cobalt resin in DM, with a yield of ~ 1.3 mg protein/L culture. The major band on Coomassie Blue -stained SDS-PAGE had an apparent size ~70 kDa, consistent with the predicted molecular mass of 73.7 kDa for the tagged protein lacking a signal sequence, together with minor bands of higher and lower apparent molecular mass (Fig 6B). Most of the latter, like the ~70 kDa band, is also stained with antibodies against the His tag, suggesting that they corresponded to oligomeric forms and degradation products of CBZIP respectively (Fig 6B).

Constructs encoding simple C-terminally His-tagged forms of *E*. *coli* NupC did not express at all. We therefore turned to vectors pL40, pL41, pL53 and pL55 (Fig 2) to express and purify both *E*. *coli* NupC and its homologue VcCNT from *V*. *cholerae*.

Both the double *Strep*-tag II and the HisMBP constructs gave equivalent expression levels (Fig 6C for double *Strep*-tag II, not shown for MBP). We chose to focus on expression using the HisMBP constructs, as the resin needed for purification costs less. The level of expression of the HisMBP constructs depended on the promoter: both NupC and VcCNT expressed better from a T7 promoter than expression from a ptac promoter (Fig 6E: the pL55 plasmid). We thus used protein expressed from the pL55 plasmids for further purification. These express either NupC or VcCNT under the T7 promoter and with an N-terminal HisMBP HRV tag. For NupC, the pL55 plasmid was compared with the pL41 plasmid expressing the double *Strep*-tag II NupC construct. We could purify and remove the affinity tags by protease digestion (Fig 6). TEV efficiently cleaved off the double *Strep*-tag II, so after cleavage, anti-Strep antibody no longer detects purified NupC while the *Strep*-tag II tagged TEV protease is still detected (Fig 6D), and we could remove the TEV protease completely by a second Streptactin purification step, where the purified cleaved protein, which is in the flow through, is not detectable by an antibody against the *Strep* tag II sequence (Fig 6D). Similarly, both the pL55-expressed NupC and VcCNT were cleaved efficiently by the His-HRV protease, and the cleaved protein is in the flow through of the second Ni-NTA purification (Fig 6F).

### Activity of CBZIP and CNTs

We used activity assays to determine whether these proteins, with both termini extracellular, were correctly folded and inserted into the membrane. For CBZIP, its ability to transport zinc was measured as for AXZIP, following reconstitution of the purified protein into proteoliposomes. As expected, the rate of Zn^2+^ uptake into proteoliposomes harbouring the purified protein was substantially greater than for protein-free liposomes (S2A Fig). We also tested uptake of Cd^2+^ and Ni^2+^ using the same assay but with CdCl_2_ or NiCl_2_ at a final concentration of 2 mM (S2B and S2C Fig).

Both Ni^2+^ and Cd^2+^ enhance the fluorescence of the indicator in a fashion similar to that of Zn^2+^, indicating that they represent alternative substrates for CBZIP. We could not measure the transport of Mg^2+^ nor of Mn^2+^ directly, because neither substantially enhanced the fluorescence of FluoZin™-1 (data not shown). However, at a concentration of 2 mM neither metal ion decreased the fluorescence response of MntH2 proteoliposomes containing FluoZin™-1 to 2 mM ZnCl_2_ for a period of 2 min (S2C Fig). This apparent lack of competition for uptake suggests that neither metal ion is a substrate of the transporter.

**Fig 7:**
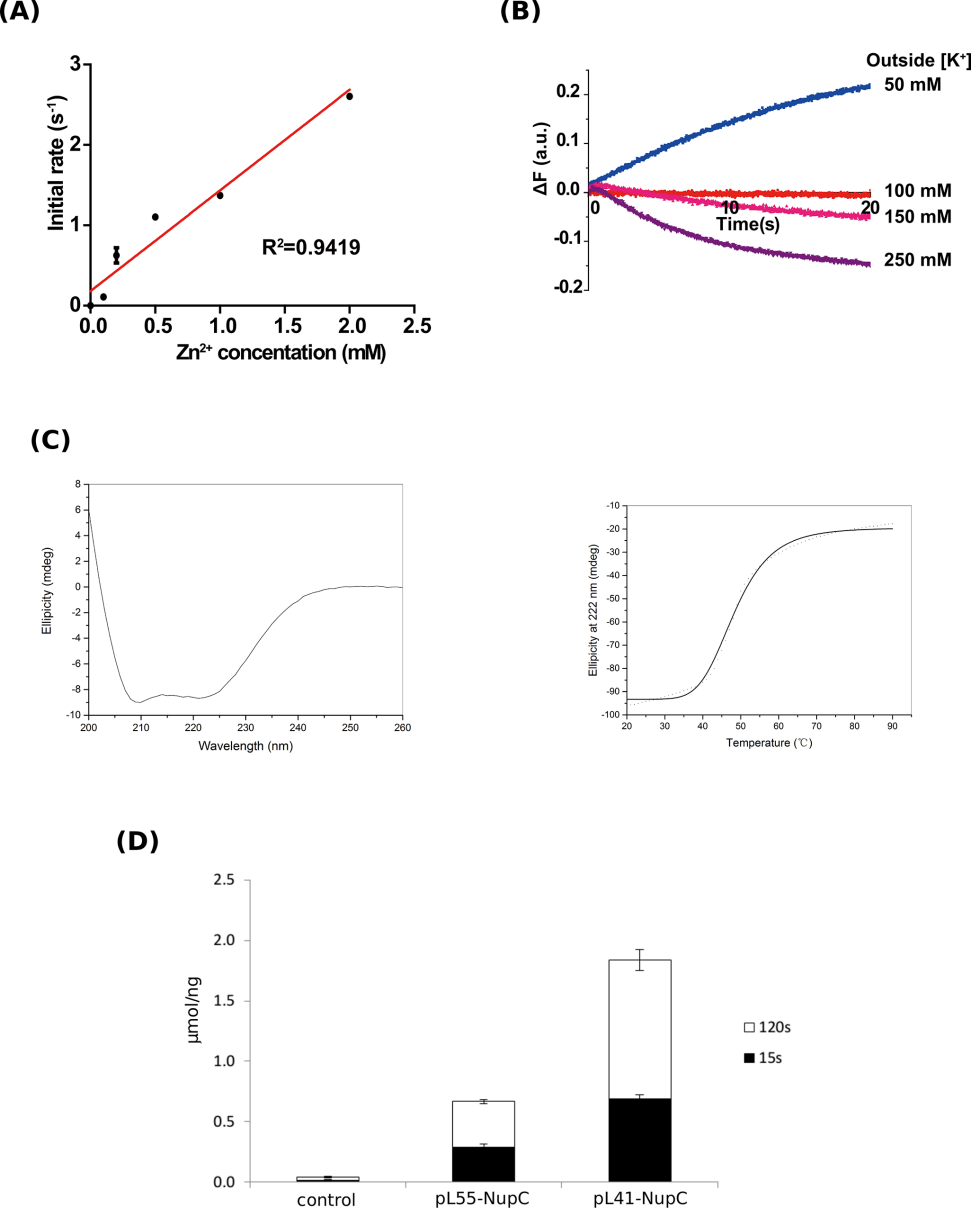
(A) Concentration dependence of Zn^2+^ uptake by proteoliposomes containing CBZIP. Fluorescent assays of zinc uptake were performed as described in Fig 5D with extravesicular Zn^2+^ concentration from 50 μM to 2 mM. (B) Membrane potentials drive bidirectional zinc fluxes. The effect on the fluorescence of encapsulated FluoZin™-1 caused by dilution of CBZIP proteoliposomes containing 100 mM K^+^ and 50 μM Zn^2+^ into medium containing 50 μM Zn^2+^ with varying K^+^ concentrations as indicated. (C) Analysis of the secondary structure of NupC expressed from construct pL55 by circular dichroism spectroscopy. The analysis of the spectrum using the SELCON3 algorithm indicated that the composition were 81.0% ± 0.2% α-helix, 0.5% ± 0.3% β-sheet and 21.4% ± 0.7% turns plus random coil. Thermal stability was measured by ramping the temperature from 10°C to 90°C, at a rate of 1°C per second. CD measurements were performed at 222 nm following each 1°C increase in temperature, and are plotted following correction for the buffer blank. The solid line shows a non-linear fit of these data to the Boltzmann equation. (D) Transport assay by NupC. Radioactively labelled Uridine import was measured on intact cells expressing NupC from either pL55 or pL40. Transport was stopped after 15s or 120s by filtration has described in material and methods. As a control, mock cells were also tested (control).

Kinetic analysis of Zn^2+^ uptake into proteoliposomes containing AXZIP suggested it may be a channel rather than a transporter (Fig 5D). As CBZIP is only distantly related to AXZIP, we wanted to determine if it likewise exhibited such channel-like behaviour, even though ZIP family proteins are designated as transporters rather than channels. We therefore measured the initial rate of zinc influx at different extravesicular zinc concentrations. The initial rate of fluorescence change depended linearly on the Zn^2+^ concentration, with no evidence of saturation up to the maximum concentration investigated (2 mM) (Fig 7A). This behaviour resembled that both of AXZIP and ZIPB [51].

We used a different set of assays to determine if the CNTs were correctly folded because there is no convenient assay for CNTs reconstituted into proteoliposomes. The CD spectra of NupC purified from pL41 (data not shown) and pL55 (Fig 7C), and of VcCNT (data not shown) all suggested that the purified proteins were correctly folded as they were essentially all α-helical: both the double *Strep*-tag II or and HisMBP tag allowed expression and purification of folded transporters. We also verified if the tagged proteins were functional before purification by studying the transport activity of StrepNupC (pL41), HisMBP NupC (pL55) (Fig 7D) and HisMBP VcCNT (not shown) in whole cells (Fig 7D). It is clear that the double *Strep*-tag II NupC (pL41-NupC) is almost three times more active than HisMBP NupC (pL55-NupC) (Fig 7D). Nonetheless, the HisMBP tagged protein expressed from a T7 promoter seems to be better suited for further biophysical studies because the expression level of the protein is greater (Fig 6).

## Discussion

Historically, proteins with either one or two termini in the periplasm have proven difficult to purify due to probable alteration of expression and/or folding of proteins by the conventional poly-histidine tags. We decided to investigate this issue by using a combination of strains, vectors, tags and proteases to yield pure and functional proteins suitable for biophysical studies. The proteins chosen have important biological activities and have either one or both termini in the periplasm.

The first family selected is a putative Manganese transporter MntH. The promoter region of the MntH gene possesses a binding motif for the Mn^2+^-sensitive transcriptional regulator EfaR, and Mn^2+^ has been found to downregulate transcription of the gene [26], consistent with its putative function in manganese transport. Human members of this family possess a cytoplasmic N-terminal and a periplasmic C-terminal. As for the ZIP family, some submembers possess an extra TM which flips the C-terminal back in the cytosol. Although no structural information is available for MntH2, or indeed for any other member of the Nramp family, it forms part of the Pfam Clan APC (CL0062), the amino acid-polyamine-organocation superfamily of transporters [52]. The structures of several such transporters have been solved, revealing them to possess duplicated inverted domains, each with five TM α−helices [52]. A model of MntH2 based on the structure of the proton-coupled amino acid transporter ApcT [53] exhibits an α-helix content of 75% (data not shown), consistent with the figure of 81.0% α-helix revealed by CD spectroscopy of the purified protein. Protein purification to homogeneity and demonstration of its monodispersity by SEC now opens up the way for more detailed examination of the MntH2 structure by X-ray crystallography and other approaches.

The ZIP family members we have investigated belong to two distinct subfamilies: subfamily II (CBZIP) and the GufA subfamily (PFZIP and AXZIP). Although PFZIP could not be purified in an active form, AXZIP and CBZIP were successfully expressed, purified and reconstituted in proteoliposomes. Both proteins are able to mediate significantly higher zinc influx compared to empty liposomes. Additionally, preliminary data suggest that CBZIP mediates bidirectional transport of zinc, depending on the membrane potential (Fig 7B). This is in agreement with the channel activity found for ZipB, a bacterial member of the GufA subfamily, and confirms that the ZIP family contains both transporters and channels.

Similarly, following the same methodology and despite their N_out_-C_out_ topology, the concentrative nucleoside transporters (NupC and VcCNT) were purified and shown to be to be folded, stable and active. In addition, we determined that the best expression-tag combination is an N-terminally His-MBP-HRV-3C tagged protein expressed from a T7 promoter. The HRV-3C protease has the advantage over the TEV protease in that it can work at 4°C and in the presence of a wide variety of detergents [54]. It is thus more suitable for membrane protein purification. The choice of expression strains and growing media conditions (temperature/media) should however be adapted to each membrane protein as this can vary from one to the other.

This paper describes the development of a series of new vectors and general strategies for the expression and purification of various membrane proteins where either one or both termini are extracellular. We show that by using a limited combination of tags and proteases (TEV or HRV-3C), we can purify five out of six proteins to homogeneity in an active, folded form. We believe that these tools and approaches will prove generically useful in the production of prokaryote membrane proteins for subsequent biochemical and biophysical studies.

## Supporting Information

S1 Fig Phylogenetic tree of ZIP family members. All family members from *A*. *thaliana* (At, green), *H*. *sapiens* (Hs, blue) and *S*. *cerevisiae* (Sc red) are included, together with examples of eubacterial family members (magenta), archaeal family members (orange) and a second example of a fungal Zip I subfamily member. UniProt accession numbers are as follows: HsZIP1, Q9NY26; HsZIP2, Q9NP94; HSZIP3, Q9BRY0; HsZIP4, Q6P5W5; HsZIP5, Q6ZMH5; HsZIP6, Q13433; HsZIP7, Q92504; HsZIP8, Q9C0K1; HsZIP9, Q9NUM3; HsZIP10, Q9ULF5; HsZIP11, Q8N1S5; HsZIP12, Q504Y0; HsZIP13, Q96H72; HsZIP14, Q15043; AtIRT1, Q38856; AtIRT2, O81850; AtIRT3, Q8LE59; AtZIP1, O81123; AtZIP2, Q9LTH9; AtZIP3, Q9SLG3; AtZIP4, O04089; AtZIP5, O23039; AtZIP6, O64738; AtZIP7, Q8W246; AtZIP8, Q8S3W4; AtZIP9, O82643 (corrected for misidentification of start codon); AtZIP10, Q8W245; AtZIP11, Q94EG9; AtZIP12, Q9FIS2; AtIAR1, Q9M647; AtZTP29, Q940Q3; ScZRT1, P32804; ScZRT2, Q12436; ScZRT3, P34240; ScATX2, Q12067; ScYKE4, P40544; EnZIP (*Emericella nidulans*) Q5AZR4; CBZIP (*Coxiella burnettii*), Q83BJ7; RsZIP (*Ralstonia solanacearum*) B5S1Z4; MxGufA (*Myxococcus xanthus*) Q06916; EcZupT (*Escherichia coli*), P0A8H3; ZIPB (*Bordetella bronchiseptica*), Q7WJT8; PFZIP (*Pseudomonas fluorescens*), Q4K7I2; GSZIP (*Geobacter sulfurreducens*), Q74GP0; DVZIP (*Desulfovibrio vulgaris*), Q72WF3; AXZIP (*Achromobacter xylosoxidans*), V9S2P1; MlZIP (*Methanofollis liminatans*), J1L0Y0; FpZIP (*Ferroglobus placidus*), D3RXQ7.
(TIF)

S2 Fig Zn2+ uptake by proteoliposomes containing MntH2. Three fluorescent repeats of zinc uptake were performed as described in Fig 5D with extravesicular Zn2+ concentration of 1 mM. (B) Comparison of Zn2+ uptake by empty proteoliposomes (red line), NupC-(green line) or MntH2-(Blue line) containing proteoliposomes in the presence of 250 μM Zn2+ as compared to uptake by MntH2- containing proteoliposomes (Black line) in the absence of 0 uM Zn2+.
(TIFF)

S3 Fig CBZIP transport properties of divalent cation (A) Comparison of zinc uptake by protein-free liposomes and proteoliposomes containing CBZIP. Liposomes (control) and proteoliposomes (CBZIP) containing 200 μM FluoZin™-1 were mixed with zinc-containing assay buffer to yield a final [Zn^2+^] of 2 mM, and the resultant fluorescence changes recorded using a stopped flow fluorimeter. The normalized fluorescence change (ΔF/ΔF_max_) was determined by dividing the observed fluorescence change (ΔF) by that induced by adding 1% β-OG to the extravesicular medium (ΔF_max_). Stopped flow fluorescence measurements were made using an excitation wavelength of 490 nm and emission was monitored using a filter with a cut-off wavelength of 515 nm. The results shown are the means of three measurements. (B) CBZIP-mediated Ni^2+^ transport. Liposomes (control) and proteoliposomes (CBZIP) containing 200 μM FluoZin™-1 were mixed with nickel-containing assay buffer to yield a final [Ni^2+^] of 2 mM, and the resultant fluorescence changes recorded using a stopped flow fluorimeter. The normalized fluorescence change (ΔF<Ni>/ΔF_max_<Ni>) was determined by dividing the observed fluorescence change (ΔF) by that induced by adding 1% β-OG to the extravesicular medium. Stopped flow fluorescence measurements were made using an excitation wavelength of 490 nm and emission was monitored using a filter with a cut-off wavelength of 515 nm. The results shown are the means of three measurements. (C) BZIP-mediated Cd^2+^ transport. Experiments were performed the same as described in (A), except cadmium-containing assay buffer was used here. (D) Apparent effects of other divalent metal ions on CBZIP-mediated transport of 2 mM Zn^2+^. Uptake experiments on proteoliposomes reconstituted with CBZIP were initiated by adding 2 mM ZnCl_2_ (final concentration) alone or with the indicated concentrations of other divalent metal ions. Fluorescence changes (ΔF) relative to the addition of buffer alone was measured using a fluorimeter (Photon Technology International) using an excitation wavelength of 490 nm and an emission wavelength of 515 nm. The results shown are the means of three measurements ± standard deviation. The significance of the differences between the “No additive” and other samples were analyzed by “One Way ANOVA” and are indicated as “**” when p<0.01.
(TIF)

S1 Table Oligonucleotides used in this study. (DOCX)
