# Supplementary figures and images for: A Versatile Strategy for Production of Membrane Proteins with Diverse Topologies: Application to Investigation of Bacterial Homologues of Human Divalent Metal Ion and Nucleoside Transporters
Source: PLoS One. 2015 Nov 25;10(11):e0143010. doi: 10.1371/journal.pone.0143010 (PMC4659628; doi:10.1371/journal.pone.0143010)

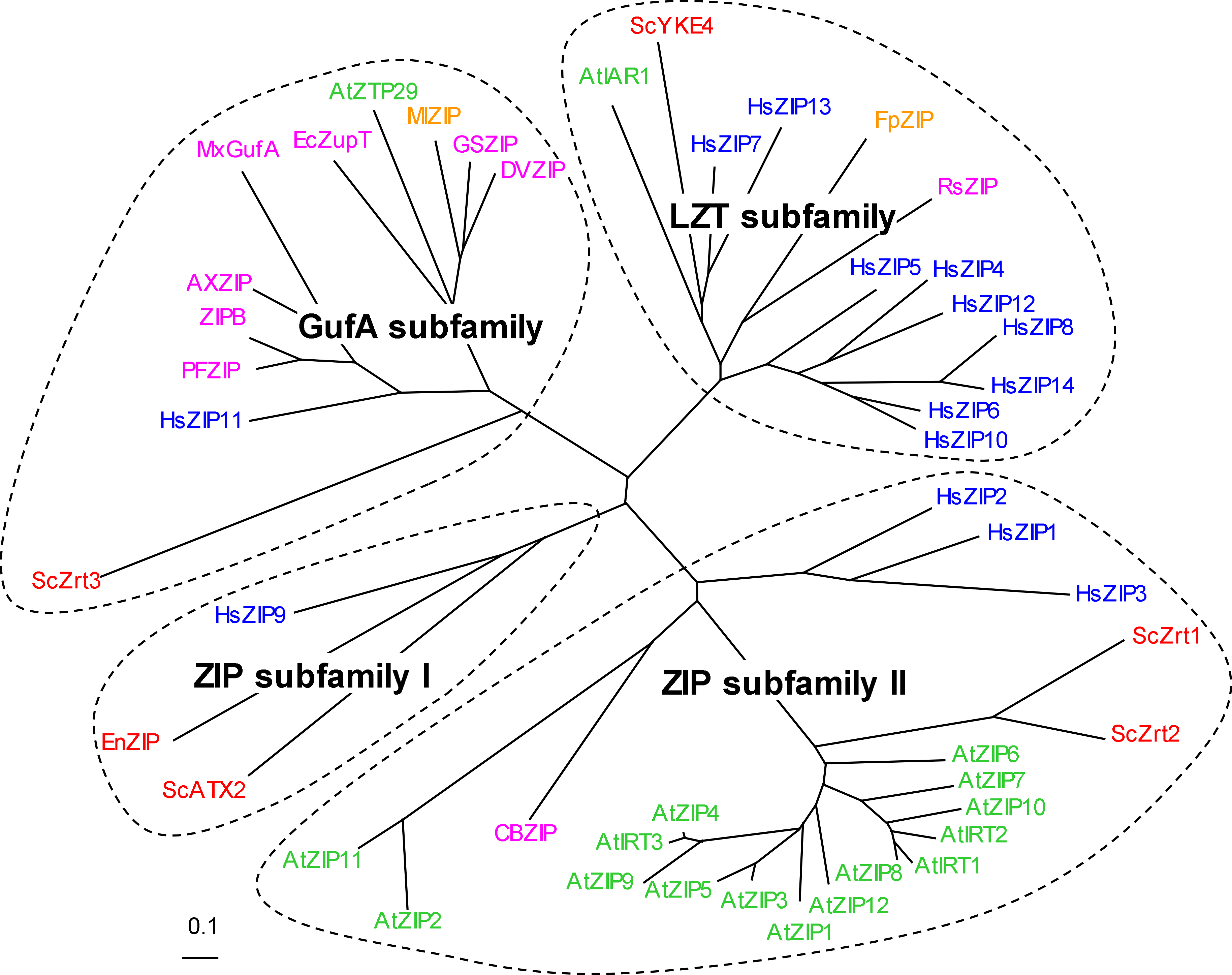

Supplement: S1 Fig — All family members from A. thaliana (At, green), H. sapiens (Hs, blue) and S. cerevisiae (Sc red) are included, together with examples of eubacterial family members (magenta), archaeal family members (orange) and a second example of a fungal Zip I subfamily member. UniProt accession numbers are as follows: HsZIP1, Q9NY26; HsZIP2, Q9NP94; HSZIP3, Q9BRY0; HsZIP4, Q6P5W5; HsZIP5, Q6ZMH5; HsZIP6, Q13433; HsZIP7, Q92504; HsZIP8, Q9C0K1; HsZIP9, Q9NUM3; HsZIP10, Q9ULF5; HsZIP11, Q8N1S5; HsZIP12, Q504Y0; HsZIP13, Q96H72; HsZIP14, Q15043; AtIRT1, Q38856; AtIRT2, O81850; AtIRT3, Q8LE59; AtZIP1, O81123; AtZIP2, Q9LTH9; AtZIP3, Q9SLG3; AtZIP4, O04089; AtZIP5, O23039; AtZIP6, O64738; AtZIP7, Q8W246; AtZIP8, Q8S3W4; AtZIP9, O82643 (corrected for misidentification of start codon); AtZIP10, Q8W245; AtZIP11, Q94EG9; AtZIP12, Q9FIS2; AtIAR1, Q9M647; AtZTP29, Q940Q3; ScZRT1, P32804; ScZRT2, Q12436; ScZRT3, P34240; ScATX2, Q12067; ScYKE4, P40544; EnZIP (Emericella nidulans) Q5AZR4; CBZIP (Coxiella burnettii), Q83BJ7; RsZIP (Ralstonia solanacearum) B5S1Z4; MxGufA (Myxococcus xanthus) Q06916; EcZupT (Escherichia coli), P0A8H3; ZIPB (Bordetella bronchiseptica), Q7WJT8; PFZIP (Pseudomonas fluorescens), Q4K7I2; GSZIP (Geobacter sulfurreducens), Q74GP0; DVZIP (Desulfovibrio vulgaris), Q72WF3; AXZIP (Achromobacter xylosoxidans), V9S2P1; MlZIP (Methanofollis liminatans), J1L0Y0; FpZIP (Ferroglobus placidus), D3RXQ7. (TIF) [file pone.0143010.s001.tif]

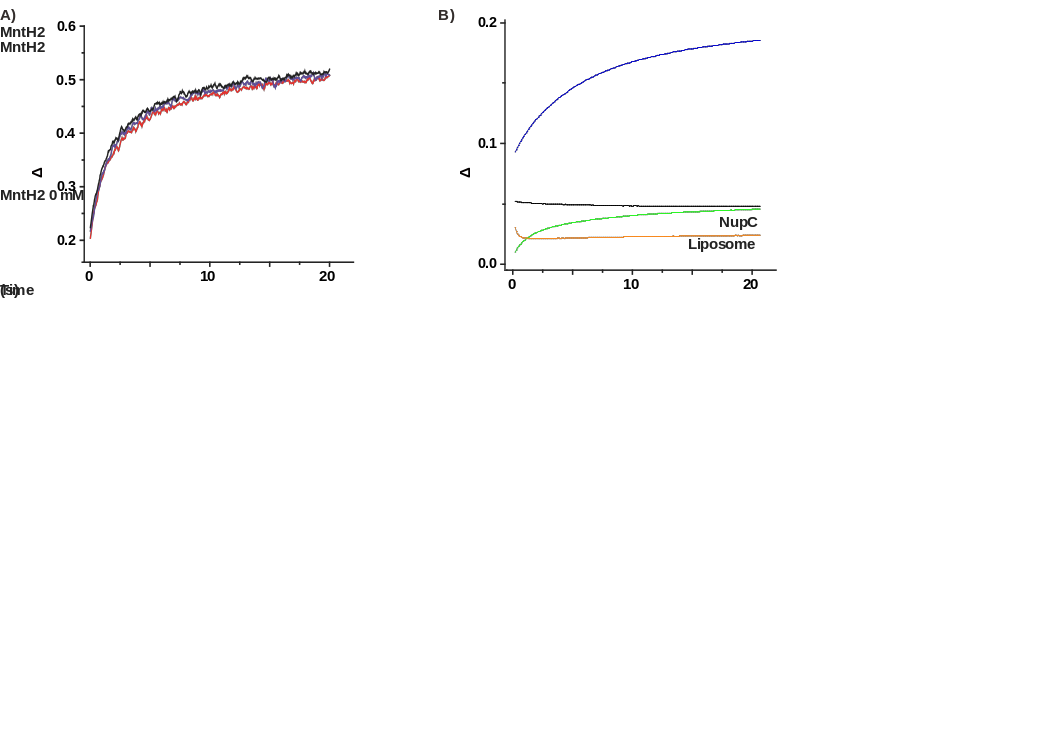

Supplement: S2 Fig — Three fluorescent repeats of zinc uptake were performed as described in Fig 5D with extravesicular Zn2+ concentration of 1 mM. (B) Comparison of Zn2+ uptake by empty proteoliposomes (red line), NupC-(green line) or MntH2-(Blue line) containing proteoliposomes in the presence of 250 μM Zn2+ as compared to uptake by MntH2- containing proteoliposomes (Black line) in the absence of 0 uM Zn2+. (TIFF) [file pone.0143010.s002.tiff]

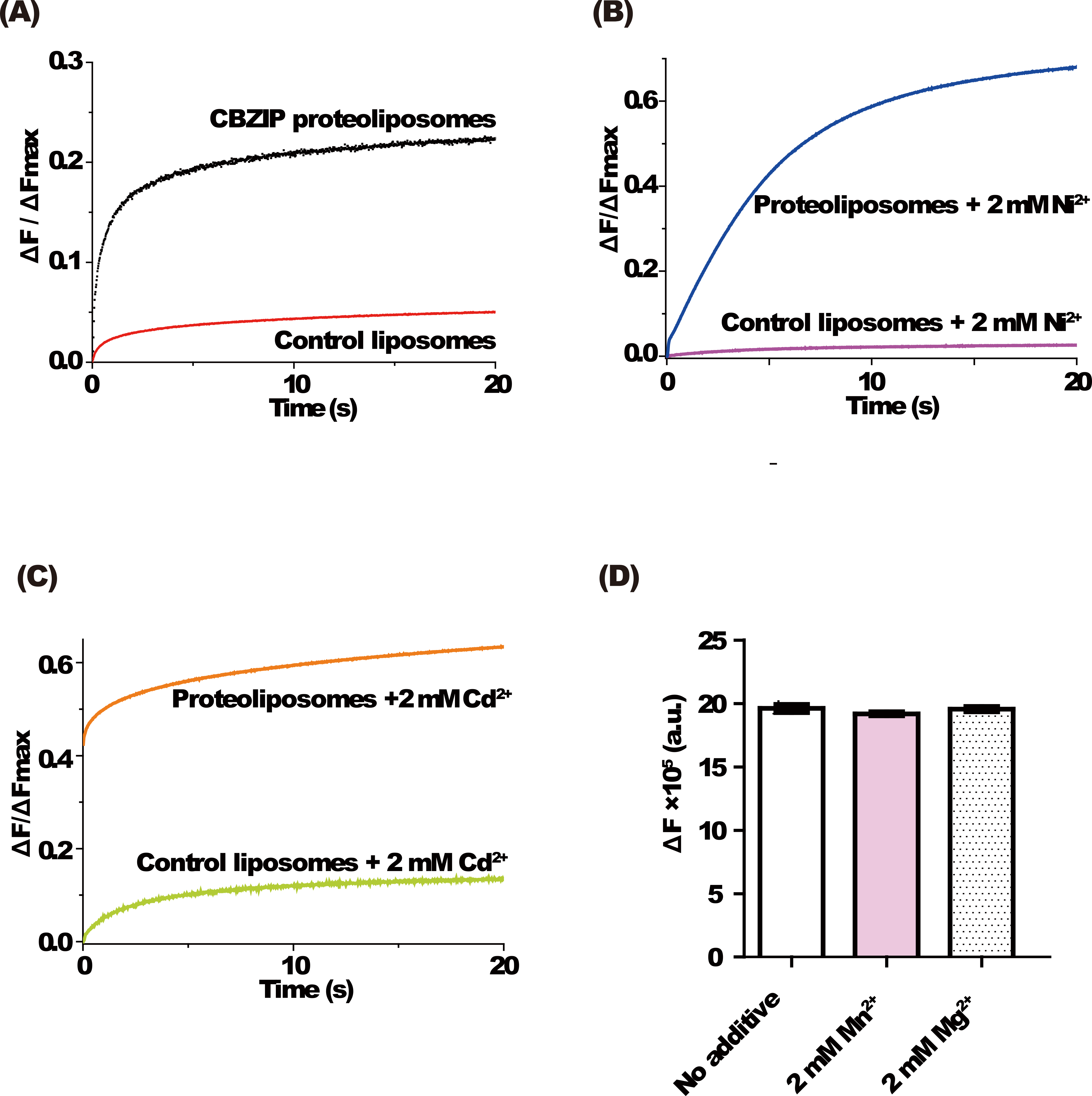

Supplement: S3 Fig — (A) Comparison of zinc uptake by protein-free liposomes and proteoliposomes containing CBZIP. Liposomes (control) and proteoliposomes (CBZIP) containing 200 μM FluoZin™-1 were mixed with zinc-containing assay buffer to yield a final [Zn2+] of 2 mM, and the resultant fluorescence changes recorded using a stopped flow fluorimeter. The normalized fluorescence change (ΔF/ΔFmax) was determined by dividing the observed fluorescence change (ΔF) by that induced by adding 1% β-OG to the extravesicular medium (ΔFmax). Stopped flow fluorescence measurements were made using an excitation wavelength of 490 nm and emission was monitored using a filter with a cut-off wavelength of 515 nm. The results shown are the means of three measurements. (B) CBZIP-mediated Ni2+ transport. Liposomes (control) and proteoliposomes (CBZIP) containing 200 μM FluoZin™-1 were mixed with nickel-containing assay buffer to yield a final [Ni2+] of 2 mM, and the resultant fluorescence changes recorded using a stopped flow fluorimeter. The normalized fluorescence change (ΔF/ΔFmax) was determined by dividing the observed fluorescence change (ΔF) by that induced by adding 1% β-OG to the extravesicular medium. Stopped flow fluorescence measurements were made using an excitation wavelength of 490 nm and emission was monitored using a filter with a cut-off wavelength of 515 nm. The results shown are the means of three measurements. (C) BZIP-mediated Cd2+ transport. Experiments were performed the same as described in (A), except cadmium-containing assay buffer was used here. (D) Apparent effects of other divalent metal ions on CBZIP-mediated transport of 2 mM Zn2+. Uptake experiments on proteoliposomes reconstituted with CBZIP were initiated by adding 2 mM ZnCl2 (final concentration) alone or with the indicated concentrations of other divalent metal ions. Fluorescence changes (ΔF) relative to the addition of buffer alone was measured using a fluorimeter (Photon Technology International) using an excitat [file pone.0143010.s003.tif]
