# Supplementary material for: A Versatile Strategy for Production of Membrane Proteins with Diverse Topologies: Application to Investigation of Bacterial Homologues of Human Divalent Metal Ion and Nucleoside Transporters
Source: PLoS One. 2015 Nov 25;10(11):e0143010. doi: 10.1371/journal.pone.0143010 (PMC4659628; doi:10.1371/journal.pone.0143010)
Supplement: S1 Table — (DOCX) [file pone.0143010.s004.docx]

**S1 Table Oligonucleotides used in this study**

| **Name, restriction site(s)** | **Sequence (5' → 3') (restriction sites underlined)** |
| --- | --- |
| *Oligonucleotides used for amplification of ORFs for vector construction* | |
| AXZIP-F; *Nhe*I | GCTAGCATGAATACCTTCAGCCGTC |
| AXZIP-R; *Eco*RI, *Sbf*I | GAATTCCCTGCAGGTCAGCCCAGCGCGGTG |
| PFZIP-F; *Spe*I | ACTAGTGCACTCTCTTCATCACCTGCAAGATCGCATC |
| PFZIP-R; *Sbf*I | CCTGCAGGACCCAAGGCGGTATCGAGAAACATCATCAC |
| MntH2-F; *Avr*II | CCTAGGAAAAATTCAGAAGAACATGAACCAAAG |
| MntH2-R; *Sbf*I | GCCTGCAGGTTTAACCTCCTCTACTTGTTGCT |
| VcCNT-F; *Nhe*I | GCTAGCAGCCTGTTTATGAGCCTCATCGGC |
| VcCNT-R; *Sbf*I | CCTGCAGGGAAAGAGAGGAAGAAGCCAGCG |
| NupC-F; *Nhe*I | GCTAGCGACCGCGTCCTTCATTTTGTAC |
| NupC-R; *Sbf*I | CCTGCAGGCAGCACCAGTGCTGCGATTGAC |
